# Investigating the Impact of the Missing Significant Objects in Scene Recognition Using Multivariate Pattern Analysis

**DOI:** 10.3389/fnbot.2020.597471

**Published:** 2020-12-17

**Authors:** Jin Gu, Baolin Liu, Weiran Yan, Qiaomu Miao, Jianguo Wei

**Affiliations:** ^1^College of Intelligence and Computing, Tianjin University, Tianjin, China; ^2^School of Computer and Communication Engineering, University of Science and Technology Beijing, Beijing, China

**Keywords:** scene recognition, significant object, semantic relationship, multivariate pattern analysis, fMRI

## Abstract

Significant objects in a scene can make a great contribution to scene recognition. Besides the three scene-selective regions: parahippocampal place area (PPA), retrosplenial complex (RSC), and occipital place area (OPA), some neuroimaging studies have shown that the lateral occipital complex (LOC) is also engaged in scene recognition processing. In this study, the multivariate pattern analysis was adopted to explore the object-scene association in scene recognition when different amounts of significant objects were masked. The scene classification only succeeded in the intact scene in the ROIs. In addition, the average signal intensity in LOC [including the lateral occipital cortex (LO) and the posterior fusiform area (pF)] decreased when there were masked objects, but such a decrease was not observed in scene-selective regions. These results suggested that LOC was sensitive to the loss of significant objects and mainly involved in scene recognition by the object-scene semantic association. The performance of the scene-selective areas may be mainly due to the fact that they responded to the change of the scene's entire attribute, such as the spatial information, when they were employed in the scene recognition processing. These findings further enrich our knowledge of the significant objects' influence on the activation pattern during the process of scene recognition.

## Introduction

Scene recognition is a common and important brain activity that can help us access environmental information. Previous studies have indicated that scene recognition relies on intrinsic properties of scenes related to 3D spatial structure, such as expanse or degree of openness (Kravitz et al., 2011; Park et al., 2011; Lowe et al., 2016), and the deepness of scene (Greene and Oliva, 2009; Amit et al., 2012; Park et al., 2015). Furthermore, it has been appreciated that the significant objects in the scene play a vital role in scene recognition. As an important part of the scene, the objects in the scene are of great significance to the scene recognition. In addition to the object size or other related properties (Cate et al., 2011; Konkle and Oliva, 2012; Bainbridge and Oliva, 2015), the scene recognition can be influenced by the association of the scene and objects (Gagne and MacEvoy, 2014; Linsley and Macevoy, 2014a; Sastyin et al., 2015). Many studies have suggested that the nature of scene recognition is the information integration of objects in the scene (Biederman, 1987; Biederman et al., 1988). Moreover, behavioral studies have shown that there is a significant decline in the recognition accuracy when objects are removed from the scene, and the classification accuracy based on brain activation pattern was lower when the objects were removed (MacEvoy and Epstein, 2011). Furthermore, there is a decrease in recognition accuracy when adding the objects that are not semantically associated with the scene (Davenport and Potter, 2004; Joubert et al., 2007).

The related neural mechanism of scene processing has received much attention in the decades. With the help of functional magnetic resonance imaging (fMRI) and other neuroimaging technologies, it has been found that there are three main areas involved in scene processing (MacEvoy and Epstein, 2011; Dilks et al., 2013; Cukur et al., 2016). Previous neuroimaging studies have indicated that the parahippocampal place area (PPA) exhibited a stronger response to scenes than the single object (Henderson et al., 2008; Persichetti and Dilks, 2016). In addition, stimuli from different scenes could activate different signal patterns in PPA (Walther et al., 2009, 2011; MacEvoy and Epstein, 2011; Epstein and Morgan, 2012), which can suggest that the PPA is associated with the processing of scene recognition. The retrosplenial complex (RSC) can be activated when people view or imagine scenes (O'Craven and Kanwisher, 2000), and it has been suggested to be responsible for spatial navigation (Henderson et al., 2008; Auger et al., 2012; Marchette et al., 2014). Moreover, different scenes could evoke different encoding information in RSC (Walther et al., 2009). The occipital place area (OPA) can respond to visually presented scenes (Hasson et al., 2003; MacEvoy and Epstein, 2007; Dilks et al., 2011). In recent years, some neuroimaging studies have found that OPA also plays a causal role in scene perception (Dilks et al., 2013; Ganaden et al., 2013; Kamps et al., 2016b).

It has been appreciated that PPA, RSC, and OPA are the key cortical regions underlying our ability to recognize scenes and use this information in navigation, however, these regions may play different roles in the specific process of scene recognition (Kamps et al., 2016a). There is still no consensus on what information can be encoded in these regions, and the neural mechanism underlying the association between significant objects and scene recognition remains to be clarified. In related studies, the PPA was demonstrated to be responsible for processing the semantic information and showed a stronger response to objects that shared a strong semantic with the scene (Aminoff et al., 2007; Hassabis et al., 2007; Bar et al., 2008; Summerfield, 2010; Howard et al., 2011). Harel et al. found that the PPA could integrate the spatial information of scenes and object information in scenes, and was sensitive to the absence or presence of both the objects and the scene (Harel et al., 2013). However, MacEvoy and Epstein argued that the lateral occipital complex (LOC) could process the semantic information between the scene and objects, which was not found in the PPA (MacEvoy and Epstein, 2011). These inconsistent findings indicate that the role of the PPA has not been definitively determined in describing the semantic association between the scene and the objects. The RSC may share similar functions in scene processing (Maguire, 2001) and one recent functional connectivity study suggested the RSC and PPA formed a scene recognition component (Hao et al., 2016). However, it is unclear whether activation on the RSC is significantly affected by the objects within the scene. Another scene-selective region OPA may prefer to deal with local scene elements rather than global scene properties (Kamps et al., 2016a), therefore we speculated the activation on the OPA would be different if the significant objects are masked in the scenes.

In addition to the above scene-selective regions, the object-selective region can take part in the scene processing and process the relationship between objects and scene. As an important area related to object recognition, the lateral occipital complex (LOC) has shown the correlation between the activity pattern induced by objects and that induced by scenes in several studies (Biederman, 1987; Malach et al., 1995; Grill-Spector et al., 1998; Kourtzi and Kanwisher, 2000; Carlson et al., 2003; James et al., 2003; Sayres and Grill-Spector, 2008; Pitcher et al., 2009). Scenes could be decoded by the activity pattern of objects that had strong semantic relationships with scenes (Peelen et al., 2009; MacEvoy and Epstein, 2011). Furthermore, Harel et al. reported a strong decrease in neural activity when objects were removed from the scene (Harel et al., 2013). These studies indicated a relationship between the activation in the LOC [including the lateral occipital cortex (LO) and the posterior fusiform area (pF)] and significant objects within the scene.

To study the brain neural mechanism of the association between significant objects and scene recognition, we designed an experiment with the scene-categorization task including behavioral and fMRI procedure which has been reported in the work of Miao et al. (2018). In the experiments, four types of images were shown to the participants, with each type including intact images of the scene and images after removing one or two significant objects from the scene. By masking the object in the scene picture, we can study the effect of objects in scene recognition. In our previous study, Miao et al. calculated the signal change and performed functional connectivity analysis based on the object-selective region LOC, and the results showed that the masking objects affected the brain network during scene recognition. In the present study, we focused on the influence of masking objects on the regions of interests (ROIs) in scene recognition by the multivariate pattern analysis (MVPA). In the neuroimaging research, the method has been implemented in various studies (Haxby et al., 2001; Norman et al., 2006; Harrison and Tong, 2009). Compared with the univariate analysis, MVPA can extract multidimensional information more adequately (Norman et al., 2006; Davis et al., 2014). Combined with appropriate classification algorithms, MVPA can be used to classify activity patterns according to stimuli categories (Harrison and Tong, 2009; Emmerling et al., 2016). Therefore, MVPA shows more advantages when we want to study the spatial patterns across multiple brain states. Considering the high dimensional and small sample size of fMRI data, the Support Vector Machine (SVM) algorithm has been widely used in the brain states classification, which has shown a significant advantage in the classification performance and robustness (LaConte et al., 2005; Mourao-Miranda et al., 2005). In recent studies, cross-classification (classifiers trained on data from a condition and tested on data from the other condition or vice versa) has been applied to analyze the brain activity in different conditions (Albers et al., 2013; Boccia et al., 2017), which can contribute to comparing these related but not identical brain activities. For example, cross-classification between perception and imagery was performed to study their association and difference (Cichy et al., 2012; Vetter et al., 2014).

In the present study, we studied the influence of significant objects by the change of scene recognition capacity in some ROIs. To the effects of significant objects on related brain regions, we conducted the univariate analysis to research the signal change on the ROIs and used MVPA to analyze the activation patterns change when the significant objects were masked in the scene. We speculated that the LO and pF were mainly involved in processing the semantic correlation between the scene and significant objects in the scene, which caused the object-selective regions were sensitive to the absence of significant objects. Since the scene-selective regions play different roles in scene recognition, we speculated that the masked significant objects may have different effects on the PPA, RSC, and OPA. By the above analysis, there would be a further understanding of the significant objects' influence on the activation pattern during the process of scene recognition.

## Materials and Methods

### fMRI Data

In this paper, the fMRI dataset is from our previous study published by Miao et al. (2018). Here, we gave a brief description of the fMRI experiment for a better understanding, and more details could be found in their publication.

Fourteen right-hand healthy participants were recruited in a visual experiment, in which they watched four kinds of scenes images: kitchen and bathroom (indoor scenes), intersection, and playground (outdoor scenes). Each kind of scene had four versions (NM: complete photographs; M1(A): photographs where only object A was masked; M1(B): photographs where only object B was masked; M2: photographs where both object A and object B were masked). Defining the combination of each type of scene and masking degree as a condition, the photographs can be divided into 16 conditions (4 scene categories × 4 versions) when considering the scene category and masked degree.

**Figure 1:**
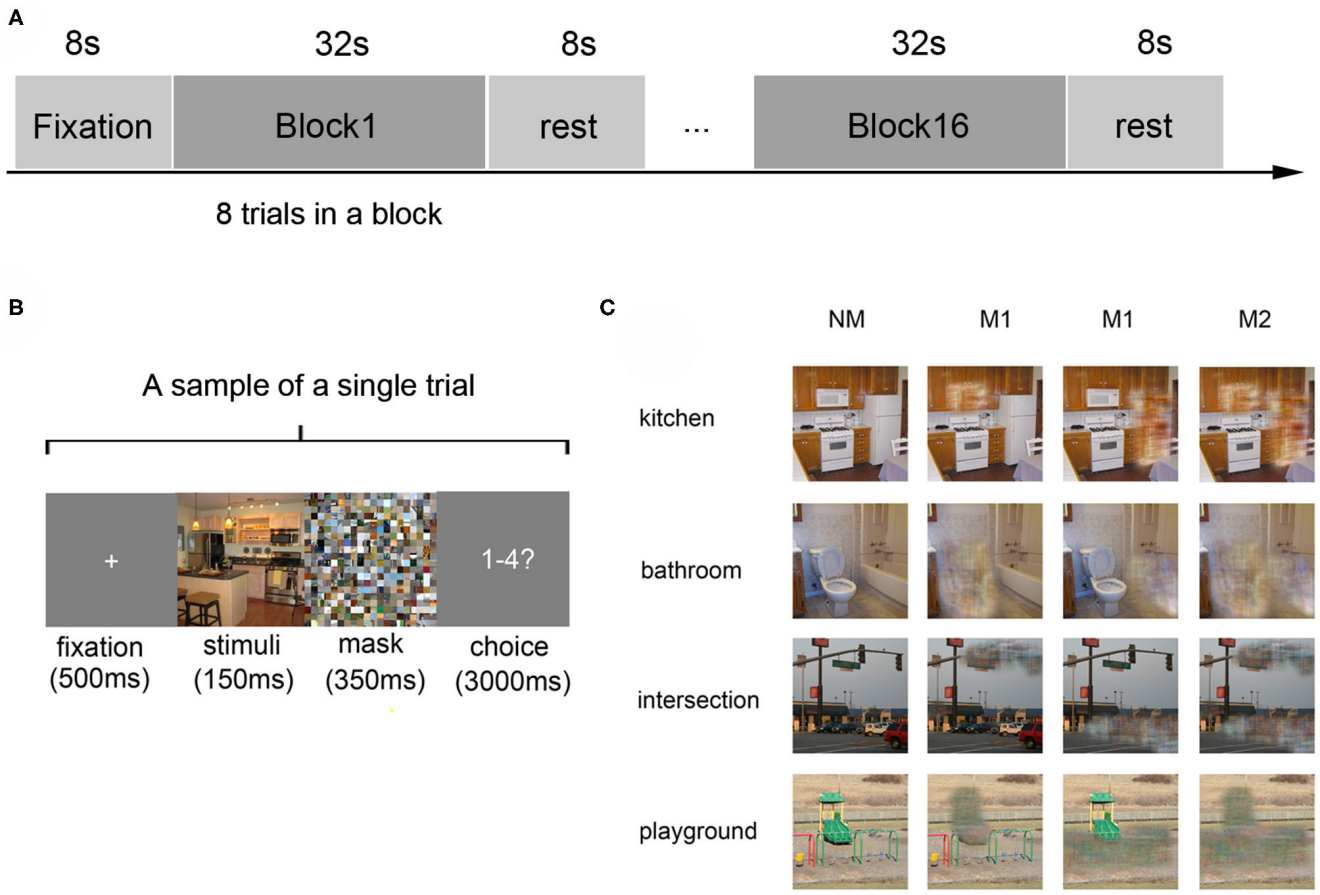
Experiment design and materials. **(A)** The experimental paradigm of a single run: the run started with an 8-s fixation and followed by 16 blocks, which were separated by an 8-s rest interval. Each block consists of 8 trials with images of the same scene category. **(B)** The paradigm of a single trial: a 500 ms fixation was shown at first, then a 150 ms stimulus image, followed by a 350 ms mask. Subjects were then required to judge the scene category within 3000 ms. **(C)** Stimulus examples from all kinds of scene categories: four kinds of scenes images in the conditions of no object was masked (NM), only one object was masked (M1), and two objects were masked (M2).

A block-design paradigm was used in this study. The experiment consisted of three functional runs and one localizer run. In the three functional runs, photographs of all the 16 different conditions were presented, in which 8 s of baseline was first presented, followed by 16 blocks. The sample object images and experimental design were shown in Figure 1, which referenced to the fMRI data description of Miao et al. (2018). In addition, the localizer run referred to the paradigm designed by MacEvoy (MacEvoy and Epstein, 2011) which could help us define the related brain areas. Then the details of fMRI data preprocessing can be found in the part of experimental procedures in the publication of Miao et al. (2018).

### ROI Selection

**Figure 2:**
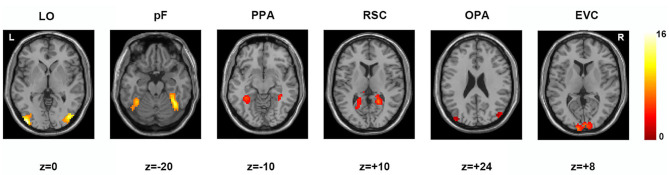
Locations of functional ROIs based on the group analysis in the localizer run. LO, lateral occipital complex; pF, posterior fusiform area; PPA, parahippocampal place area; RSC, retrosplenial complex; OPA, occipital place area; EVC, early visual cortex.

According to previous studies on the localization method of visual ROI (Malach et al., 1995; Grill-Spector et al., 1998), ROIs were defined based on the activation diagram from the localizer run. Compared with our previous work (Miao et al., 2018), we added four new ROIs, including the pF, LO, OPA, and EVC. The pF and LO were defined as through the contrast object minus phase-scrambled objects, we set the peak response in the later-ventral occipitotemporal cortex, and we located the pF and LO, respectively. OPA was defined in the transverse occipital sulcus where the response to scenes condition was stronger than objects condition. As a control area, the EVC was defined in the posterior occipital lobe through the contrast of phase-scrambled objects minus intact objects. The PPA and RSC were defined in the posterior parahippocampal-collateral sulcus region and retrosplenial cortex, referring to the work of Miao et al. (2018). Figure 2 showed the activation in each of ROI at the group level. The peak coordinates and peak intensity of functional ROIs at the group level were shown in Table 1. To avoid the interindividual differences in anatomic locations of the regions, the ROIs were identified as spheres with an 8 mm radius in every subject and the ROI coordinates for each subject were located defined at the local maxima closest to the group peak coordinates.

**Table 1:** The peak coordinates and peak intensity of functional ROIs in the group level.

**Functional ROIs**	**MNI coordinates**			**Peak intensity**
	**x**	**y**	**z**	
Left LO	−39	−90	−3	15.02
Right LO	42	−81	0	12.44
Left pF	−36	−52	−23	10.58
Right pF	39	−52	−20	12.55
Left PPA	−33	−43	−8	5.64
Right PPA	36	−40	−11	4.69
Left RSC	−15	−49	16	4.87
Right RSC	21	−49	10	6.05
Left OPA	−39	−85	28	2.02
Right OPA	48	−76	28	3.36
Left EVC	−9	−103	13	8.45
Right EVC	9	−103	16	11.85

### Multivariate Pattern Analysis

To clarify the roles of significant objects in scene recognition in ROIs, we calculated the scene classification accuracy using the activation pattern in these regions (LO, pF, PPA, RSC, and OPA) and the control area EVC. The defined ROIs contribute to feature selection, and the voxels involved in the defined ROIs were included in the classification analysis. Brain patterns are labeled according to all the scene conditions (scene categories × mask conditions). In the present study, the SVM classifier was used to implement the four-way classification (kitchen, bathroom, intersection, and playground) in different mask conditions (NM, M1, and M2) (Chang and Lin, 2011). Unsmoothed functional data were used in the classification processing because the spatial smoothing would destroy some useful variables (Samuel Schwarzkopf and Rees, 2011). The classification was conducted through the leave-one run out cross-validation approach, which contributed to the stable and dependable classification results by avoiding the higher correlation of the data from the same run (Wolbers et al., 2011; Axelrod and Yovel, 2012). Afterward, we conducted the one-sample *t*-test on the classification performances to test whether the statistical value was statistically significant (*p* < 0.05). Then the one-way repeated measures ANOVA was performed to test the effect of mask conditions. In order to assure the classification performances were reliable, we shuffled the labels and randomly assigned to the training samples and performed the four scene classification analysis with the same procedure based on the “shuffled” data (Stelzer et al., 2013).

In addition, we performed the cross-classification analysis in which the intact (NM) scene data were used to train the SVM model, and M1 and M2 data were used as the test dataset. Then, the one-sample *t*-test was performed on the classification results to show the difference and similarity of activation pattern between the complete scene and scene with masked significant objects.

### Univariate Analysis

The result of pattern recognition reflected the overall pattern of all voxels in the same region, while percent signal change can reflect the activation intensity in a single region. We used the marsbar software (http://marsbar.sourceforge.net/) to calculate the signal change in the ROIs in three masked conditions separately (NM, M1, and M2). Then paired samples *t*-test was conducted between the conditions in each region to investigate whether the activation is different across different masked conditions. In addition, we performed repeated-measures ANOVA to study the difference of activation intensity across ROIs.

## Results

### Classification Analysis by MVPA

#### Scene Classification in Different Mask Condition

To study the influence of masked objects on the scene classification, we calculated and compared the classification accuracy of the activation pattern in the three kinds of mask conditions (NM, M1, and M2). When we classified scenes based on the activation pattern in the LO, the results showed that the classification accuracy is not significant higher than the chance level (25%) when there was object masked in the scene picture (NM: 49.44%, *t*_13_ = 8.13, *p* < 0.001; M1: 21.83%, *t*_13_ = −2.75, *p* = 0.017; M2: 27.12%, *t*_13_ = 0.89, *p* = 0.391). One-way repeated-measures ANOVA was performed to test the role of mask conditions in the accuracy decline, and the result showed that the classification differences in different conditions were statistically significant (*F* = 35.69, *p* < 0.001). The *post-hoc* tests showed accuracy in NM was significantly >M1 (*p* < 0.001) and M2 (*p* < 0.001), however, there wasn't a significant difference between M1 and M2 (*p* = 0.085).

Then we performed the above analysis on other ROIs, and the activation pattern corresponding to scenes with masked objects was not significantly classified. The results showed greater classification accuracies in the NM than the conditions with masked objects.

In the pF, the successful classification was only observed in the NM condition (NM: 47.73%, *t*_13_ = 6.85, *p* < 0.001; M1: 24.63%, *t*_13_ = −0.24, *p* = 0.818; M2: 26.90%, *t*_13_ = 1.12, *p* = 0.283), and there was significant difference in the different mask conditions (*F* = 23.49, *p* < 0.001) by the one-way repeated measures ANOVA. The *post-hoc* tests showed classification accuracy in NM condition was significantly >M1 (*p* < 0.001) and M2 (*p* < 0.001). We also compared the classification accuracies in the M1 and M2, however, there wasn't significant difference (*p* = 0.406).

There was also a decline for the accuracies of scene classification in the PPA with more objects masked in three conditions (NM: 46.02%, *t*_13_ = 6.39, *p* < 0.001; M1: 25.48%, *t*_13_ = 0.40, *p* = 0.693; M2: 27.79%, *t*_13_ = 1.49, *p* = 0.160). One-way repeated measures ANOVA showed significant differences in three conditions (*F* = 26.28, *p* < 0.001) and accuracy in NM was significantly >M1 and M2 (*p* < 0.001) by *post-hoc* tests. However, there wasn't significant difference between M1 and M2 (*p* = 0.289).

In the RSC, the decoding only succeed in NM condition (NM: 49.93%, *t*_13_ = 8.59, *p* < 0.001; M1: 23.79%, *t*_13_ = −1.05, *p* = 0.311; M2: 27.75%, *t*_13_ = 1.26, *p* = 0.229), and the accuracy differences in the different mask conditions were statistically significant (*F* = 41.16, *p* < 0.001). The difference was from the classification accuracy in the NM and that in the M1 and M2 (*p* < 0.001) by *post-hoc* tests, however, there wasn't significant difference between M1 and M2 (*p* = 0.182). The classification results in the OPA were similar, that is, classification accuracies were significantly above the chance level in the NM conditions (NM: 49.18%, *t*_13_ = 9.96, *p* < 0.001; M1: 24.48%, *t*_13_ = −0.40, *p* = 0.697; M2: 29.72%, *t*_13_ = 2.04, *p* = 0.063). There was significant difference in the different mask conditions (*F* = 41.56, *p* < 0.001), and *post-hoc* tests showed NM was significantly >M1 and M2 (*p* < 0.001). However, there wasn't significant difference between M1 and M2 (*p* = 0.054).

**Figure 3:**
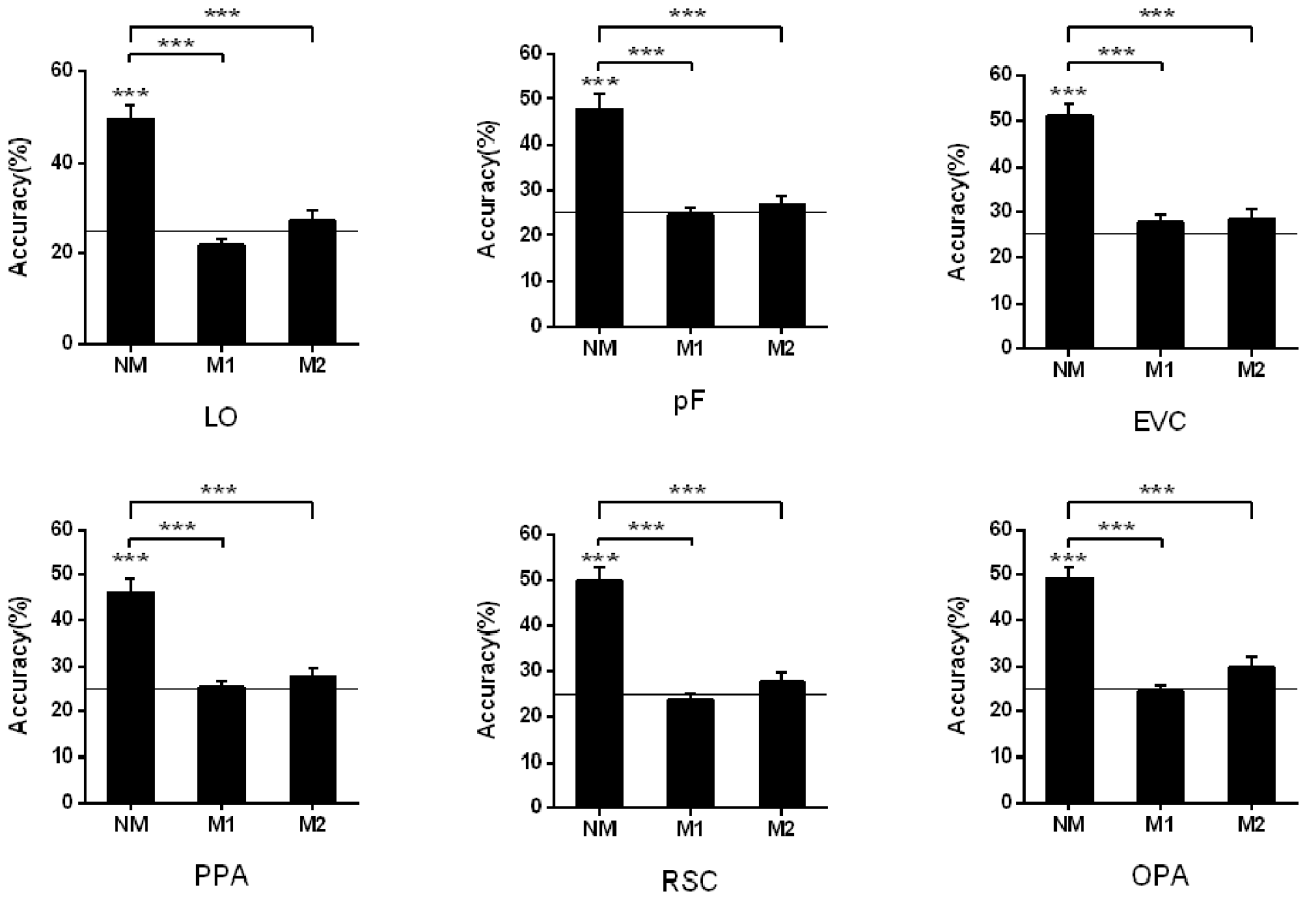
Pattern classification analysis based on the activation in ROIs. The black line indicates the chance level (25%). Stars indicate significant classification accuracy in each condition and the significant difference between the two conditions; ***p < 0.001, **p < 0.01, *p < 0.05.

In the EVC, the successful classifications were observed in the NM (NM: 51.15%, *t*_13_ = 9.65, *p* < 0.001; M1: 27.85%, *t*_13_ = 1.72, *p* = 0.110; M2: 28.61%, *t*_13_ = 1.80, *p* = 0.096). One-way repeated measures ANOVA showed significant differences in three conditions (*F* = 28.70, *p* < 0.001). The classification accuracy in the NM was significantly >M1 (*p* < 0.001) and M2 (*p* < 0.001) by *post-hoc* tests, however, there wasn't significant difference between M1 and M2 (*p* = 0.803). The classification results in all ROIs were shown in Figure 3.

#### Cross-Classification Between Different Conditions

**Figure 4:**
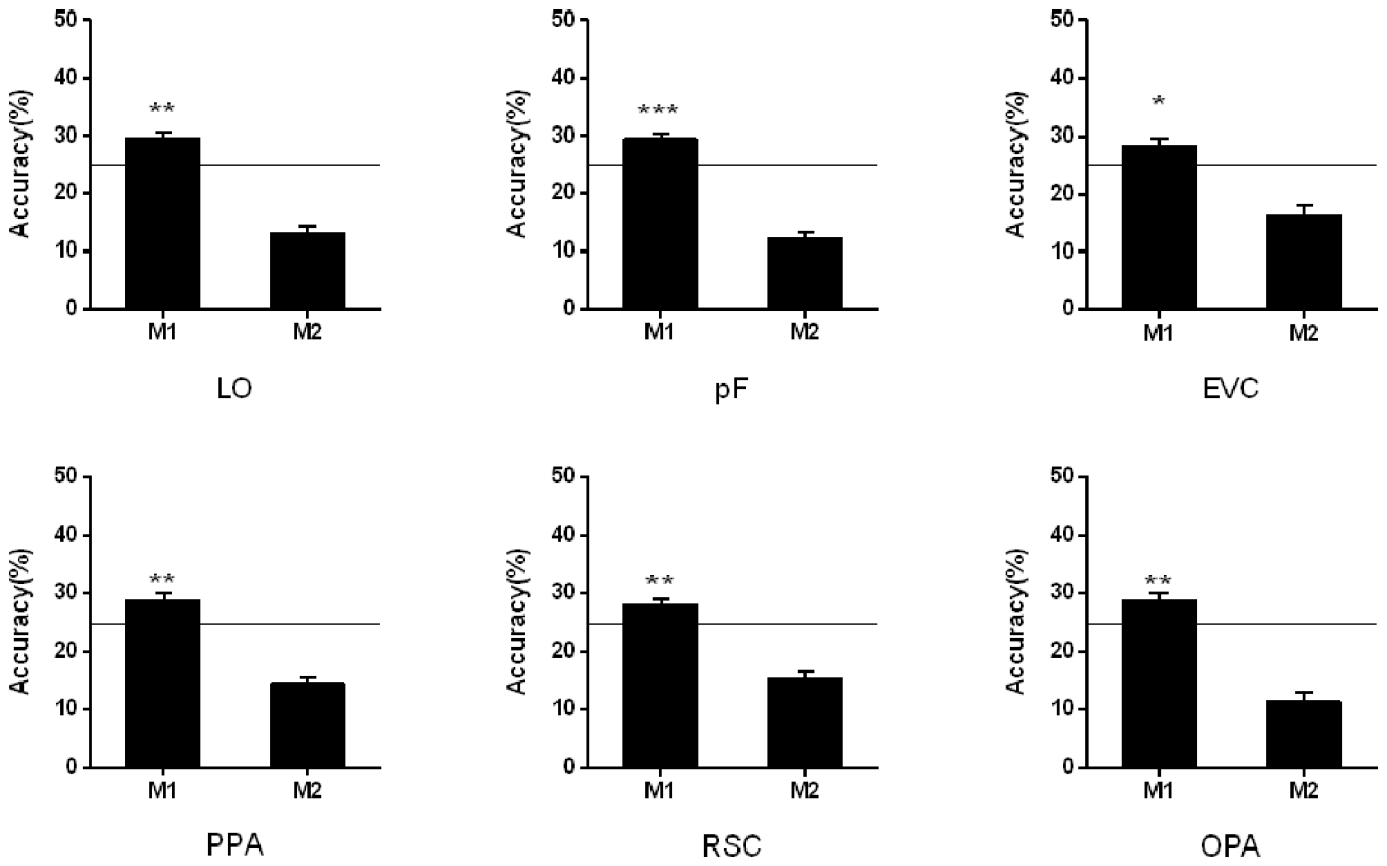
Cross-classification analysis in M1 and M2 conditions. The black line indicates the chance level (25%). Stars indicate significant classification accuracy which is higher than the chance level in each condition; ***p < 0.001, **p < 0.01, *p < 0.05.

In the cross-classification analysis, the brain data from M1 condition can be decoded when we used the data from NM as the train data. In the objects-selective regions and scene-selective regions, the classification accuracies were significantly higher than chance-level (LO: 29.56%, *t*_13_ = 3.85, *p* = 0.002; pF: 29.48%, *t*_13_ = 2.38, *p* < 0.001; PPA: 28.83%, *t*_13_ = 3.20, *p* = 0.007; RSC: 28.03%, *t*_13_ = 3.22, *p* = 0.007; OPA:28.83% *t*_13_ = 3.09, *p* = 0.009; EVC:28.42%, *t*_13_ = 2.67, *p* = 0.019). However, the classification failed in the M2 condition when we used the classification model trained on the NM condition. The cross-classification results in the M1 and M2 conditions were shown in Figure 4.

### Signal Changes in the Univariate Analysis

In addition to analyzing the changes in activation patterns, we also compared the intensity of activation in these brain regions under different conditions. During the process of scene recognition, there were significant signal changes in the LO, pF, PPA, and the control region EVC relative to the baseline state. The significant signal change and trend of reduction can be found in the LO (NM: 0.48, *t*_13_ = 4.25, *p* < 0.001; M1: 0.37, *t*_13_ = 4.27, *p* < 0.001; and M2: 0.36, *t*_13_ = 3.237, *p* = 0.006). The paired *t*-tests indicated that the signal change of NM was significantly greater than that of M1 (*t*_13_ = 2.63, *p* = 0.021) and M2 (*t*_13_ = 2.250, *p* = 0.043). The similar results can be found in the pF with significant signal changes in all the conditions (NM: 0.31, *t*_13_ = 6.06, *p* < 0.001; M1: 0.27, *t*_13_ = 8.38, *p* < 0.001; and M2: 0.22, *t*_13_ = 8.00, *p* < 0.001). There is also a significant difference between NM and M2 (*t*_13_ = 2.33, *p* = 0.037, paired *t*-test). These results are consistent with the signal change results of Miao et al. (2018), in which the LOC was studies as a whole.

In the PPA, the significant signal change was observed (NM: 0.12, *t*_13_ = 4.57, *p* < 0.001; M1: 0.10, *t*_13_ = 4.28, *p* < 0.001; and M2: 0.12, *t*_13_ = 3.34, *p* < 0.01), but paired *t*-tests indicated that there was no obvious reduction trend across different mask conditions. The results of signal change in the RSC and PPA were reported in the work of Miao et al. The results of signal change in the RSC and OPA were not significant, and we added some detailed description about the value of signal change in present study (RSC: NM: −0.012, *t*_13_ = −1.03, *p* = 0.320; M1: −0.017, *t*_13_ = −1.98, *p* = 0.070; and M2: −0.020%, *t*_13_ = −1.51, *p* = 0.154; OPA: NM: 0.12, *t*_13_ = 2.10, *p* = 0.06; M1: 0.02, *t*_13_ = 0.36, *p* = 0.73; and M2: 0.12, *t*_13_ = 1.65, *p* = 0.12).

**Figure 5:**
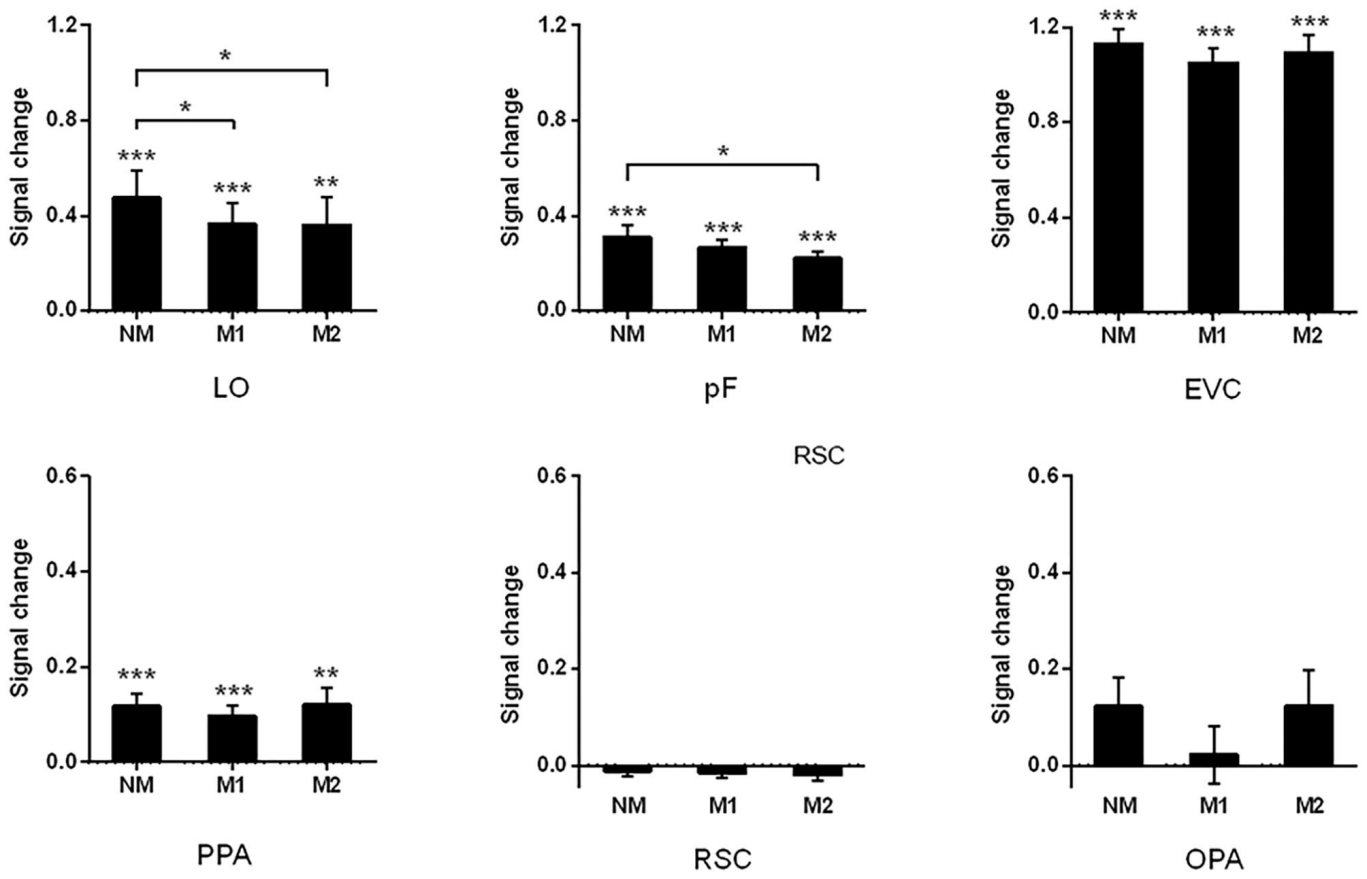
Signal changes of all ROIs with different objects are masked. The significant signal change can be found in LO, pF, EVC, and PPA. The difference between three mask conditions was observed by paired-sample *t*-test; ***p < 0.001, **p < 0.01, *p < 0.05.

In the control area EVC, significant signal change was observed in each condition (NM: 1.13, *t*_13_ = 8.61, *p* < 0.001; M1: 1.05, *t*_13_ = 9.90, *p* < 0.001; and M2: 1.09, *t*_13_ = 8.68, *p* < 0.001). Paired *t*-tests suggested that there was no significant transformation trend in the different mask conditions. The results of all ROIs were shown in Figure 5.

In addition to examining the differences in the activation intensity under different conditions, we also compared the signal changes across ROI in the same condition. The repeated-measures ANOVA was performed and the results showed that there were significant differences across ROIs in each condition (NM: *F* = 69.66, *p* < 0.001; M1: *F* = 73.01, *p* < 0.001; and M2: *F* = 66.00, *p* < 0.001). The signal change in EVC was significantly higher than that in other ROIs in each of the conditions (*p* < 0.001, *post-hoc* tests). The signal change in the LO was not significantly different from that in the pF, while there is a significant difference compared to scene-selective ROIs in conditions of NM and M1 (NM: LO vs. PPA, *p* = 0.004, LO vs. RSC, *p* = 0.004, and LO vs. OPA, *p* = 0.022; M1: LO vs. PPA, *p* = 0.021, LO vs. RSC, *p* = 0.002, and LO vs. OPA, *p* = 0.014). In the condition of M2, there is a significant difference between the LO and RSC (*p* < 0.001), which is similar to the comparison between pF and RSC (*p* < 0.001). In conditions of NM and M1, signal change in the pF was significantly higher than that in the PPA and RSC (NM: pF vs. PPA, *p* = 0.021, pF vs. RSC, *p* < 0.001; M1: pF vs. PPA, *p* = 0.029, pF vs. RSC, *p* < 0.001).

## Discussion

The objects in the scene play an important role when people identify the scene category. In the present study, we investigated the neural mechanism of this phenomenon based on the change of the brain activation pattern when participants viewed the scenes in which the significant objects were masked. Miao et al have reported the significant influence of masking objects on behavior results that the accuracy of scene recognition decreased as more masked objects were removed, while the reaction time of the participants increased (Miao et al., 2018). Our analysis of the brain activation pattern also showed the neural representation activated by different mask conditions was significantly different in the related brain regions, which suggested that significant objects played an important role in the processing of scene recognition. Furthermore, the activation pattern showed more difference when more objects were masked, and the details are different in these brain regions.

As the object-selective region, the LOC plays important role in processing object information, which has been suggested in many previous studies, but the relationship between the LOC and scene recognition remains to be further demonstrated (Biederman, 1987; Malach et al., 1995; Grill-Spector et al., 1998; Carlson et al., 2003; James et al., 2003; Sayres and Grill-Spector, 2008; Pitcher et al., 2009). In the present study, significant classification accuracy was found in the NM condition in the object-selective region, which was significantly higher than those in M1 and M2 conditions. In the control region, however, the activation intensity of EVC was not significantly different in the three conditions. Therefore, we speculated that the decline of the activation intensity in the LO and pF suggested that the object-selective region was responsible for processing object information. One recent study found that a virtual scene with a single object induced stronger activations than a scene without objects (Harel et al., 2013), which is in line with our study. Harel's work studied the virtual scene with only one object, but real scenes are more complex and there are different objects in a scene. In our study, we further investigated whether it can induce a change in the activation pattern in brain regions if different numbers of objects were masked in the scene. According to the difference of the percent signal change across the different scene conditions, we can speculate that the activation intensity in the LOC had an intimate relationship with the amount of within-scene objects. According to classification analysis, the activation pattern of the complete scene was more effective when it was used to decode the scene categories compared with that in which one or two objects were masked in LO and pF. Statistical results showed that the accuracy of classification was not significantly different from the chance level in both of the M1 and M2 conditions, and the classification accuracies in M1 and M2 conditions were significantly lower than that in NM. Furthermore, the cross-classification succeeded between the NM and M1 conditions rather than between the NM and M2, which is consistent with the behavioral results that the more masked objects lead to the lower the scene discrimination accuracy. These results suggested there was a similar activation pattern when only one object was masked compared with the condition M2. According to the related research, we speculated that object-selective region could process the integrated information of objects and scenes in the processing of scene recognition (MacEvoy and Epstein, 2011; Linsley and MacEvoy, 2014b; Lescroart et al., 2015), and the LO and pF were responsible for processing the semantic association between objects in scenes and scenes during the processing of scene recognition.

For the scene-selective regions, the activation intensity did not change significantly across different conditions. There were significant signal changes in the PPA, however, it shows no significant difference when objects were masked. Compared with the object-selective region, the results suggested the activation intensity in the PPA was not sensitive to the univariate modulation caused by the masked object. Compared with the univariate analysis, the MVPA can contribute to finding more information based on the activation pattern. The classification accuracy was significantly higher than chance-level in the PPA under the condition of NM, however, the decoding failed in the conditions of M1 and M2. The result showed that the masked object made an effect on the activation pattern of PPA, although there was no significant difference in the activation intensity. In the scene recognition, the spatial properties of scenes may be more dominant for the PPA. Previous studies suggested that the PPA was only responsible for the attribute information of scenes, and it could not be activated by objects(MacEvoy and Epstein, 2011; Persichetti and Dilks, 2016), however, the other studies argued that the PPA was capable of processing the objects' information related to the scene (Aminoff et al., 2007; Hassabis et al., 2007; Henderson et al., 2008; Summerfield, 2010; Harel et al., 2013) and it could even be affected by the information of significant objects (Linsley and Macevoy, 2014a), which was consistent with our results. Also, there was a study that confirmed the structural and functional basis of the PPA on processing the scene-relevant relationship between objects and scenes by using the method of voxel-based morphometry and functional connectivity (Hao et al., 2016).

In the activation intensity analysis, it was revealed that the signal changes were not significant in the RSC and OPA in full scenes or scenes with masked objects. As a control region, it is conceivable that EVC activation intensity must be significant. We speculated that the stimuli were presented for participants a relatively short time, which made the scene-selective regions acquire no enough information on the scene attribute. Furthermore, these regions contribute more to the spatial navigation and imagination instead of scene categorization (Epstein, 2008; Marchette et al., 2014; Vass and Epstein, 2016), which may be another reason for not finding significant signal change. Although the signal change did not show a significant value, the full scene could still be decoded based on the activation pattern of the RSC and OPA. Based on the classification results, we found that only full scenes could be successfully decoded in the RSC, while the scenes with masked objects could not be classified. The RSC was demonstrated to be specifically responsible for processing the spatial information of scenes (Henderson et al., 2008). One recent study indicated that the RSC anchored to local topographical features and generalized across local spatial contexts with similar structures (Marchette et al., 2014). Moreover, the absence of objects might affect the representation of the entire attribute of the scene space. When one or two objects were removed, the spatial structure of the scene was damaged, and the activation pattern could not provide enough information, which caused the low accuracy of pattern recognition. Therefore, we speculated that RSC is not recruited to process the semantic associations between scenes and objects, which is consistent with another research of our group about scene processing (Wang et al., 2018). According to recent studies, the OPA may pay more attention to local scene elements rather than global scene properties (Kamps et al., 2016a). We speculated that the masked objects in the scene might change the local elements so the OPA is sensitive to the lack of objects. These findings indicated that activation in the RSC and OPA was more responsive to the properties of the scene. As a control region, the MVPA results of the EVC shared more similarities with the scene-selective regions. The scene classification succeeded in the NM condition, and there was a significant decline in the two masked scene conditions. The EVC process low-level features properties in visual processing (such as the presence of text). We speculated that scene-selective areas are recruited to process scene recognition based on the entire attribute of the scene while the object-selective region can process the semantic association between objects and scenes in the processing of scene recognition.

## Conclusions

In this study, we tried to explore the roles and mechanisms of related regions in processing the association between the significant objects and scene by masking different objects in the scene. The impact of significant objects in scene recognition has been demonstrated in previous behavior research. The present study suggested that the LO and pF were sensitive to the absence of significant objects, and the effect may be caused by processing the semantic correlation between the scene and significant objects in the scene. The scene-selective regions were sensitive to the masked objects which might be due to the change of local scene elements, which caused the difference in spatial properties of the scene. Overall, the regions related to the scene recognition were affected by the information of significant objects according to their main function in the scene recognition, and the significant objects in the scene could play an important role in scene recognition.

## Data Availability Statement

The datasets presented in this article are not readily available because the data is being a part of an ongoing study in the research group so the datasets cannot be shared at this time. Requests to access the datasets should be directed to the corresponding author.

## Ethics Statement

The studies involving human participants were reviewed and approved by the Research Ethics Committee of Tianjin Key Lab of Cognitive Computing and Application, Tianjin University. The patients/participants provided their written informed consent to participate in this study.

## Author Contributions

BL and WY designed the experiments. JG and WY analyzed results. JG wrote the main manuscript text and prepared the figures. BL, QM, and JW contributed to the manuscript revision. All authors contributed to discuss the results and have approved the final manuscript.

## Conflict of Interest

The authors declare that the research was conducted in the absence of any commercial or financial relationships that could be construed as a potential conflict of interest.
